# Development and Validation of a Model for Predicting the Risk of Acute Kidney Injury Associated With Contrast Volume Levels During Percutaneous Coronary Intervention

**DOI:** 10.1001/jamanetworkopen.2019.16021

**Published:** 2019-11-22

**Authors:** Chenxi Huang, Shu-Xia Li, Shiwani Mahajan, Jeffrey M. Testani, Francis P. Wilson, Carlos I. Mena, Frederick A. Masoudi, John S. Rumsfeld, John A. Spertus, Bobak J. Mortazavi, Harlan M. Krumholz

**Affiliations:** 1 Center for Outcomes Research and Evaluation, Yale New Haven Hospital, New Haven, Connecticut; 2 Section of Cardiovascular Medicine, Department of Internal Medicine, Yale School of Medicine, New Haven, Connecticut; 3 Program of Applied Translational Research, Department of Internal Medicine, Yale School of Medicine, New Haven, Connecticut; 4 Division of Cardiology, University of Colorado Anschutz Medical Campus, Aurora; 5 Saint Luke’s Mid America Heart Institute, Department of Cardiology, University of Missouri, Kansas City; 6 Department of Computer Science and Engineering, Texas A&M University, College Station; 7 Department of Health Policy and Management, Yale School of Public Health, New Haven, Connecticut

## Abstract

**Importance:**

Determining the association of contrast volume during percutaneous coronary intervention (PCI) with the risk of acute kidney injury (AKI) is important for optimizing PCI safety.

**Objective:**

To quantify how the risk of AKI is associated with contrast volume, accounting for the possibility of nonlinearity and heterogeneity among different baseline risks.

**Design, Setting, and Participants:**

This prognostic study used data from the American College of Cardiology National Cardiovascular Data Registry CathPCI Registry for 1694 US hospitals. Derivation analysis included 2 076 694 individuals who underwent PCI from July 1, 2011, to June 30, 2015. Validation analysis included 961 863 individuals who underwent PCI from July 1, 2015, to March 31, 2017. Data analysis took place from July 2018 to May 2019.

**Exposure:**

Contrast volume during PCI.

**Main Outcomes and Measures:**

Acute kidney injury was defined using 3 thresholds for preprocedure to postprocedure creatinine level increase (ie, ≥0.3 mg/dL, ≥0.5 mg/dL, and ≥1.0 mg/dL). A model quantifying the association of contrast volume with AKI was developed, and the existence of nonlinearity and heterogeneity were examined by likelihood ratio tests. The model was derived in the training set (a random 50% of the derivation cohort), and performance was evaluated in the test set (the remaining 50% of the derivation cohort) and an independent validation set by area under the receiver operating characteristic curve (AUC) and calibration slope of observed vs predicted risks.

**Results:**

The 2 076 694 patients in the derivation set had a mean (SD) age of 65.1 (12.1) years, and 662 525 (31.9%) were women; 133 306 (6.4%) had creatinine level increases of at least 0.3 mg/dL, 66 626 (3.2%) had creatinine level increases of at least 0.5 mg/dL, and 28 378 (1.4%) had creatinine level increases of at least 1.0 mg/dL. In the validation set of 961 843 patients (mean [SD] age, 65.7 [12.1] years; 305 577 [31.8%] women), these rates were 62 913 (6.5%), 34 229 (3.6%), and 15 555 (1.6%), respectively. The association of contrast volume and AKI risk was nonlinear (χ^2^_26_ = 1436.2; *P* < .001) and varied by preprocedural risk (χ^2^_20_ = 105.6; *P* < .001). In the test set, the model yielded an AUC of 0.777 (95% CI, 0.775-0.779) for predicting risk of a creatinine level increase of at least 0.3 mg/dL, 0.839 (95% CI, 0.837-0.841) for predicting risk of a creatinine level increase of at least 0.5 mg/dL, and 0.870 (95% CI, 0.867-0.873) for predicting risk of a creatinine level increase of at least 1.0 mg/dL; it achieved a calibration slope of 0.998 (95% CI, 0.989-1.007), 0.999 (95% CI, 0.989-1.008), and 0.986 (95% CI, 0.973-0.998), respectively, for the AKI severity levels. The model had similar performance in the validation set (creatinine level increase of ≥0.3 mg/dL: AUC, 0.794; 95% CI, 0.792-0.795; calibration slope, 1.039; 95% CI, 1.030-1.047; creatinine level increase of ≥0.5 mg/dL: AUC, 0.845; 95% CI, 0.843-0.848; calibration slope, 1.063; 95% CI, 1.054-1.074; creatinine level increase of ≥1.0 mg/dL: AUC, 0.872; 95% CI, 0.869-0.875; calibration slope, 1.103; 95% CI, 1.089-1.117).

**Conclusions and Relevance:**

The association of contrast volume with AKI risk is complex, varies by baseline risk, and can be predicted by a model. Future research to evaluate the effect of the model on AKI is needed.

## Key Points

**Question:**

What is the association of contrast volume used during percutaneous coronary intervention with the risk of acute kidney injury?

**Findings:**

In this prognostic study of nearly 3 million adults undergoing percutaneous coronary intervention, a predictive model for the association of contrast volume with the risk of acute kidney injury was developed and validated. The association was nonlinear along the spectrum of contrast volume and varied according to patient baseline risk.

**Meaning:**

This model could support personalized approaches that consider the complex association between contrast volume and the risk of acute kidney injury when determining contrast dosing during interventional procedures.

## Teaser

This prognostic study quantifies the association of contrast volume during percutaneous coronary intervention with the risk of acute kidney injury, accounting for the possibility of nonlinearity and heterogeneity among different baseline risks.

## Introduction

Acute kidney injury (AKI) is a common and serious complication of percutaneous coronary intervention (PCI) that is associated with an increased risk of death and higher costs of care. To inform personalized risk-mitigation interventions for patients undergoing PCI, researchers and clinicians have sought accurate risk estimations using the patients’ baseline characteristics.^1^ Reducing contrast volume during PCI is considered the most actionable and effective intervention.^2^ Various studies support this approach, with lower rates of AKI among individuals who receive lower contrast volumes.^3,4,5^

While limiting contrast volume is an acknowledged method of reducing AKI risk, clinicians need to balance reducing contrast volume with providing adequate contrast to permit coronary evaluation and treatment.^6^ Thus, there is a need to better understand how AKI risk varies continuously with increasing contrast volume. Furthermore, among patients with different baseline risks, the association between contrast volume and AKI risk may differ and may not be linear across the spectrum of contrast volume. Identification of such heterogeneity and nonlinearity in risk could potentially improve clinical decision-making for individual patients. However, few studies have quantified the change in AKI risk based on differences in contrast volume, and fewer have examined potential heterogeneity in the association of contrast volume with AKI as a function of patient baseline risk of AKI. Moreover, studies on AKI risk estimation have exclusively considered a binary AKI definition, focusing on a single severity level. Providing risk estimates for multiple severities of AKI, especially for more serious AKIs that are associated with higher rates of morbidity and mortality,^7^ could facilitate better informed decisions on intervention.

Accordingly, we developed a prediction model for AKI to determine the association of contrast volume with AKI risk in the National Cardiovascular Data Registry (NCDR) CathPCI Registry. Building on results of a previously developed machine learning model for estimating patients’ preprocedural risk of AKI,^8^ we employed a generalized additive model (GAM) to accommodate the potential nonlinear associations between contrast volume and AKI risk and the heterogeneity of this association among different risk groups.

## Methods

### Data Source and Study Population

The NCDR CathPCI Registry has been described elsewhere.^9,10^ This study included PCIs performed from July 1, 2011, to June 30, 2015 (2 710 866 procedures) (eFigure 1 in the Supplement). We applied the same exclusion criteria used in developing the NCDR AKI model.^1^ We excluded PCIs that were not the first during a single hospitalization (n = 72 065), that had same-day discharge (n = 161 271), that were missing serum creatinine levels before or after the procedure (n = 324 438), and that were performed on patients who were already on dialysis (n = 60 523). Additionally, we excluded PCIs that were missing contrast volume measurements (n = 7209). We further excluded patients with preprocedural creatinine levels of 0.3 mg/dL or less (n = 2247) or higher than 4.0 mg/dL (n = 6419) (to convert creatinine levels to micromoles per liter, multiply by 88.4); the decision-making process in the care of these patients is different from that used for other patients,^11^ and including them in the analyses could mask the association between AKI and contrast volume for other patients. The final analytic cohort included the remaining 2 076 694 PCI procedures. To ensure the generalizability of the derived model for prospective use, we validated our results in a cohort that included 961 843 PCI procedures from July 1, 2015, to March 31, 2017, by implementing the same inclusion and exclusion criteria. The Yale University institutional review board approved the study and waived the patient informed consent requirement for the use of retrospective data from the NCDR registry. This study followed the Transparent Reporting of a Multivariable Prediction Model for Individual Prognosis or Diagnosis (TRIPOD) reporting guideline.

### Study Outcome

The primary outcome was AKI, defined by the difference between preprocedure and postprocedure creatinine levels. We considered a spectrum of AKI severity, defined by the following 3 thresholds for absolute creatinine level increase: at least 0.3 mg/dL, at least 0.5 mg/dL, and at least 1.0 mg/dL. This definition was different from the staging criteria used by the Acute Kidney Injury Network (AKIN)^12^ and the Kidney Disease Improving Global Outcomes.^13^ Stage 2 and stage 3 definitions from AKIN or Kidney Disease Improving Global Outcomes resulted in an event rate less than 1% in the study cohort, which was not sufficient to produce a useful risk-stratification model. The thresholds used in this study were gradations within stage 1 AKI. Previous studies have shown that modest increases in creatinine levels within stage 1 AKI are independently associated with short-term and long-term adverse clinical outcomes and increased length of hospitalization.^14,15,16^

### Risk Factors

We used preprocedural AKI risk and contrast volume as risk factors to develop the model for guiding contrast dosing. The preprocedural risk was stage 1 AKI, defined according to AKIN as the risk of a creatinine increase of at least 0.3 mg/dL or 150% of the preprocedural level. This risk estimate was used to characterize patients’ baseline risk and was calculated from a previously developed prediction model.^8^ This prediction model was derived from an earlier cohort from the NCDR CathPCI Registry (with procedures from June 1, 2009, to June 30, 2011) using gradient descent boosting and 13 variables. The detailed definitions and coding of these variables can be found in eTable 1 in the Supplement. Missing values of these baseline variables were imputed by the mode for categorical variables and by the median for continuous variables.

### Statistical Analysis

The distribution of contrast volume used for patients with different preprocedural risks was summarized as the number and percentage of patients using varying contrast volumes. The pattern of contrast volume usage among individual patients was also shown in 2-dimensional scatterplots. The scatterplots were colored by observed AKI risks estimated by the event rate among each patient’s 250 nearest neighbors, which were determined by the Mahalanobis distance in preprocedural risk and contrast volume.

To derive and validate the model, we randomly divided the final analytic cohort into a training set (50% of the cohort) and a test set (the remaining 50%). The model was built using the training set, and the performance of the model was evaluated on both the test set and an independent temporal validation set.

We used a GAM to model and predict the association of contrast volume with AKI risk for different patients. To enable modeling for the 3 severity levels of AKI, we converted the creatinine changes into 4 mutually exclusive categories (ie, <0.3 mg/dL; 0.3 to <0.5 mg/dL, 0.5 to <1.0 mg/dL, and ≥1.0 mg/dL) as the dependent variable and used the logit of the preprocedural risk and the contrast volume as the predictors, employing a multinomial logit link function. The GAM was capable of learning complex associations via a sum of nonlinear functions and was therefore useful in uncovering nonlinear associations and interactions.^17^ For this study, the nonlinear functions were the tensor products of cubic regression splines of the 2 predictors. Cubic regression splines and tensor products were used to model potential nonlinearity interactions of the predictors. To assess the existence of nonlinearity and heterogeneity in the association of contrast volume with AKI, we further derived 3 alternative prediction models, as follows: (1) linear terms of the 2 predictors, (2) linear predictors plus an interaction term, and (3) nonlinear predictors with no interaction term. The fitness of these models to the data was compared by likelihood ratio tests. These models were also compared visually by 2-dimensional contour plots of the predictions against the observed risks. We also examined whether the association of contrast volume with AKI differed for different severity levels of AKI by comparing the prediction performance of the model in the training set with an alternative model using ordinal logit link function with a proportional odds assumption.

Model discrimination was evaluated by the area under the receiver operating characteristic curve (AUC), and calibration was evaluated by the calibration intercept and slope of the observed rates vs the deciles of the predicted risks. A smooth calibration plot showing model calibration for a wider range of risks was also provided using cubic spline smoothers. Prediction accuracy was assessed by the Brier score, defined as the mean squared difference between the observed outcome and predicted risks. Brier scores quantify the error made by predictions from observations. We also calculated the predictive range of the model by the difference of the observed rates in the first and tenth deciles of predicted risks. We reported the performance of the model for predicting each of the 3 AKI severities.

To demonstrate the utility of the prediction model to guide contrast dosing, we plotted the predicted AKI risks with 95% CIs as a function of contrast volume for a range of preprocedural risks. We further calculated the odds ratio (OR) and absolute risk difference (RD) of AKI by an increase of contrast volume of 200 mL along the spectrum of contrast volume. To safeguard against overextrapolation from what the data can inform, predictions were calculated for values of preprocedural risks and contrast volumes where there were at least 10 patients in the training set within the neighborhood of these values; the neighborhood was defined as a 5% difference in preprocedural risk and a 50 mL difference in contrast volume.

Model performance was further evaluated in the validation cohort, and all performance metrics in the main analyses were reported. All analyses were developed in R version 3.4.0 (the R Foundation for Statistical Computing).^18^ The GAM was performed using the mgcv version 1.8.24 R package.^19^ For all 2-tailed χ^2^ tests, *P* < .05 was considered statistically significant.

## Results

Table 1 describes event rates, characteristics, and contrast volumes for patients included in the derivation and validation sets. In the derivation set of 2 076 694 patients (mean [SD] age, 65.1 [12.1] years; 662 525 [31.9%] women), 133 306 patients (6.4%) had a creatinine level increase of at least 0.3 mg/dL, 66 626 (3.2%) had a creatinine level increase of at least 0.5 mg/dL, and 28 378 (1.4%) had a creatinine level increase of at least 1.0 mg/dL. In the validation set of 961 843 patients (mean [SD] age, 65.7 [12.1] years; 305 577 [31.8%] women), these rates were 62 913 (6.5%), 34 229 (3.6%), and 15 555 (1.6%), respectively. Compared with patients in the derivation set, patients in the validation set had higher median (interquartile range) preprocedural risk of stage 1 AKI (5.4% [3.4%-10.0%] vs 5.0% [3.2%-9.2%]) and received lower mean (SD) contrast volume (182.4 [82.3] mL vs 190.6 [86.4] mL).

**Table 1.** Study Population Characteristics

Characteristic	No. (%)	
	Derivation (n = 2 076 694)	Validation (n = 961 843)
Increase in creatinine level, mg/dL		
≥0.3	133 306 (6.4)	62 913 (6.5)
≥0.5	66 626 (3.2)	34 229 (3.6)
≥1.0	28 378 (1.4)	15 555 (1.6)
New initiation of dialysis	6351 (0.3)	3781 (0.4)
Age, mean (SD), y	65.1 (12.1)	65.7 (12.1)
Women	662 525 (31.9)	305 577 (31.8)
Race		
White	1 808 616 (87.1)	831 114 (86.4)
Black or African American	175 267 (8.4)	82 577 (8.6)
Admission source		
Emergency department	912 536 (43.9)	452 951 (47.1)
Transfer from acute-care facility	382 996 (18.4)	175 941 (18.3)
Body mass index, mean (SD)^a^	30.1 (11.1)	30.2 (9.0)
Baseline GFR, mL/min/1.73 m^2^		
Mean (SD)	78.3 (26.9)	78.3 (26.8)
≥60	1 557 373 (75.0)	721 974 (75.1)
>45 to <60	331 443 (16.0)	152 127 (15.8)
≥30 to <45	148 608 (7.2)	68 605 (7.1)
<30	39 270 (1.9)	19 137 (2.0)
Anemia	88 947 (4.3)	49 931 (5.3)
Hypertension	1 707 124 (82.2)	795 689 (82.7)
Prior MI	628 192 (30.2)	288 255 (30.0)
Prior heart failure	270 483 (13.0)	145 228 (15.1)
Prior PCI	829 374 (40.0)	379 601 (39.4)
Prior CABG	371 783 (17.9)	164 779 (17.1)
Cerebrovascular disease	265 366 (12.8)	128 059 (13.3)
Peripheral arterial disease	250 662 (12.1)	113 146 (11.8)
Chronic lung disease	324 810 (15.6)	152 343 (15.8)
Diabetes	779 247 (37.5)	376 120 (39.1)
CAD presentation		
No symptom, no angina	93 263 (4.5)	30 363 (3.2)
Symptom unlikely to be ischemic	40 761 (2.0)	16 850 (1.8)
Stable angina	250 189 (12.0)	99 456 (10.3)
Unstable angina	804 816 (38.8)	360 711 (37.5)
Non-STEMI	508 109 (24.5)	271 679 (28.2)
STEMI or equivalent	379 192 (18.3)	182 599 (19.0)
IABP before procedure	4558 (0.2)	2373 (0.2)
Heart failure within 2 wk	24 812 (11.6)	136 121 (14.2)
Cardiogenic shock within 24 h	42 717 (2.1)	21 515 (2.2)
Cardiac arrest within 24 h	45 170 (2.2)	22 009 (2.3)
Contrast volume, mL		
Mean (SD)	190.6 (86.4)	182.4 (82.3)
0 to <50	37 227 (1.8)	16 378 (1.7)
50 to <100	228 014 (11.0)	122 499 (12.7)
100 to <150	517 823 (24.9)	264 641 (27.5)
150 to <200	551 190 (26.5)	253 163 (26.3)
200 to <250	355 233 (17.1)	152 578 (15.9)
250 to <300	199 931 (9.6)	81 607 (8.5)
300 to <350	93 373 (4.5)	36 422 (3.8)
350 to <400	48 634 (2.3)	18 439 (1.9)
400 to <600	41 767 (2.0)	14 906 (1.5)
≥600	3502 (0.2)	1210 (0.1)
Preprocedural risk, median (IQR), %^b^	5.0 (3.2-9.1)	5.4 (3.4-10.0)

Abbreviations: CABG, coronary artery bypass grafting; CAD, coronary artery disease; GFR, glomerular filtration rate; IABP, intraaortic balloon pump; IQR, interquartile range; MI, myocardial infarction; PCI, percutaneous coronary intervention; STEMI, ST-segment elevation myocardial infarction.

SI conversion factor: To convert creatinine to micromoles per liter, multiply by 88.4.

^a^ Calculated as weight in kilograms divided by height in meters squared

^b^ Defined as the risk of creatinine level elevating by 0.3 mg/dL or at least 150% after PCI compared with levels measured before the procedure or initiation of dialysis.

**Figure 1.**
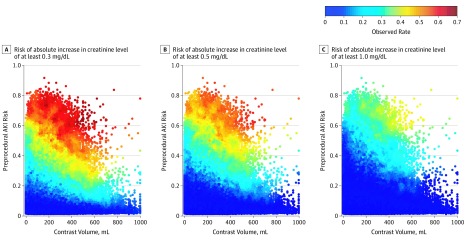
Scatterplot of Patients’ Preprocedural Acute Kidney Injury (AKI) Risks and Contrast Volumes The scatterplot was colored with observed risks of AKI estimated by each patients’ 250 nearest neighbors. To convert creatinine to micromoles per liter, multiply by 88.4.

Similar patterns of contrast volume were received by patients in different preprocedural risk groups (eTable 2 in the Supplement). For each preprocedural risk group, about half the patients received 100 to 200 mL of contrast, with the percentage gradually decreasing when contrast volume decreased to less than 100 mL or increased to greater than 200 mL. Figure 1 shows the contrast volume received by individual patients ordered by their preprocedural risk estimates and colored by the observed AKI rates. The AKI rate was higher for patients with higher preprocedural risk and generally increased with contrast volume.

**Figure 2.**
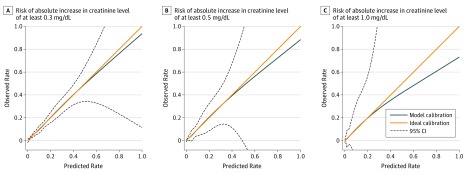
Calibration Plots of the Model Predicting Acute Kidney Injury via Splines in the Test Set Risk of absolute increase in creatinine levels was calculated in the observed vs predicted risks via cubic spline smoothing. To convert creatinine to micromoles per liter, multiply by 88.4.

Comparing the models using linear predictors vs nonlinear predictors, a test for the existence of nonlinearity was statistically significant (χ^2^_26_ = 1436.2; *P* < .001). Tests for the existence of interaction were statistically significant for comparing models using linear predictors (χ^2^_3_ = 9.1; *P* = .03) and comparing models using nonlinear predictors (χ^2^_20_ = 105.6; *P* < .001). Comparing the observed risks with predictions from different candidate models for predicting the risk of a creatinine level increase of at least 0.3 mg/dL also revealed a better fit of data to the model employing nonlinearity and interaction terms (eFigure 2 in the Supplement). Finally, the model that used an ordinal link function had worse calibration than the model using a multinomial link function (eTable 3 in the Supplement), showing the inadequacy of using the proportional odds specification to model the association of contrast volume with AKI for different severity levels. Evaluated on the test set, the best model achieved an AUC of 0.777 (95% CI, 0.775-0.779) for predicting risk of a creatinine level increase of at least 0.3 mg/dL, 0.839 (95% CI, 0.837-0.841) for predicting risk of a creatinine level increase of at least 0.5 mg/dL, and 0.870 (95% CI, 0.867-0.873) for predicting risk of a creatinine level increase of at least 1.0 mg/dL (Table 2). It achieved a calibration slope of 0.998 (95% CI, 0.989-1.007), 0.999 (95% CI, 0.989-1.008), and 0.986 (95% CI, 0.973-0.998), respectively, for the AKI severity levels. This model also had Brier scores of 0.0539 (95% CI, 0.0538-0.0540), 0.0276 (95% CI, 0.0275-0.0276), and 0.0123 (95% CI, 0.0123-0.0124), respectively, demonstrating low error and high accuracy. Good calibration between the observed and predicted risks for all 3 AKI severities are shown in eFigure 3 in the Supplement. Smooth calibration plots also show good calibration for patients (Figure 2), except for a slight overestimation toward those with higher risk. When applied to the validation set, the model achieved a similar AUC of 0.794 (95% CI, 0.792-0.795), 0.845 (95% CI, 0.843-0.848), and 0.872 (95% CI, 0.869-0.875) for the 3 AKI severities, with satisfactory calibration slopes (creatinine level increase of ≥0.3 mg/dL: 1.039; 95% CI, 1.030-1.047; creatinine level increase of ≥0.5 mg/dL: 1.063; 95% CI, 1.054-1.074; creatinine level increase of ≥1.0 mg/dL: 1.103; 95% CI, 1.089-1.117) (Table 2; eFigure 4 and eFigure 5 in the Supplement).

**Table 2.** Model Performance for Predicting Risk of Acute Kidney Injury^a^

Performance	Increase in Creatinine Level		
	≥0.3 mg/dL	≥0.5 mg/dL	≥1.0 mg/dL
**Test Set**			
Event rate, %	6.4	3.2	1.4
AUC (95% CI)	0.777 (0.775 to 0.779)	0.839 (0.837 to 0.841)	0.870 (0.867 to 0.873)
Calibration slope (95% CI)	0.998 (0.989 to 1.007)	0.999 (0.989 to 1.008)	0.986 (0.973 to 0.998)
Calibration intercept (95% CI)	0.000 (−0.001 to 0.001)	0.000 (−0.001 to 0.001)	0.000 (−0.000 to 0.000)
Brier score	0.0539 (0.0538 to 0.0540)	0.0276 (0.0275 to 0.0276)	0.0123 (0.0123 to 0.0124)
Predictive range (95% CI), %	24.5 (24.3 to 24.8)	17.0 (16.8 to 17.1)	8.6 (8.5 to 8.7)
**Temporal Validation Set**			
Event rate, %	6.5	3.6	1.6
AUC (95% CI)	0.794 (0.792 to 0.795)	0.845 (0.843 to 0.848)	0.872 (0.869 to 0.875)
Calibration slope (95% CI)	1.039 (1.030 to 1.047)	1.063 (1.054 to 1.074)	1.103 (1.089 to 1.117)
Calibration intercept (95% CI)	−0.006 (−0.007 to −0.001)	0.000 (−0.002 to −0.001)	0.000 (−0.001 to −0.000)
Brier score	0.0540 (0.0539 to 0.0542)	0.0301 (0.0301 to 0.0302)	0.0145 (0.0145 to 0.0146)
Predictive range (95% CI), %	26.7 (26.4 to 26.9)	19.2 (19.0 to 19.4)	10.3 (10.2 to 10.4)

Abbreviation: AUC, area under receiver operating characteristic curve.

SI conversion factor: To convert creatinine to micromoles per liter, multiply by 88.4.

^a^ Risk of absolute increase in creatinine of at least 0.3 mg/dL, 0.5 mg/dL, or 1.0 mg/dL.

**Figure 3.**
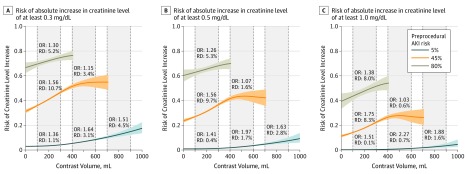
Prediction of Risks of Acute Kidney Injury (AKI) as a Function of Contrast Volume Risks of absolute increase in creatinine was calculated for patients with preprocedural AKI risk of 5%, 45%, and 80%. Odds ratios (ORs) and absolute risk differences (RDs) of AKI risks are given for increases in contrast volume from 100 to 300 mL, from 400 to 600 mL, and from 700 to 900 mL. The colored bands indicate 95% CIs. To convert creatinine to micromoles per liter, multiply by 88.4.

Figure 3 plots the estimated risks of creatinine level increases of at least 0.3 mg/dL, 0.5 mg/dL, and 1.0 mg/dL as a function of contrast volume for preprocedural risks of 5%, 45%, and 80%. The learned model (eFigure 6 in the Supplement) revealed that larger differences in risk were associated with contrast volume for patients with preprocedural risk between 25% to 75% compared with other patients. We thus chose these 3 preprocedural risks to quantify the difference in how AKI risk was associated with increasing contrast volume. For each preprocedural risk, the OR and RD of AKI were given for contrast volume increases from 100 to 300 mL, from 400 to 600 mL, or from 700 to 900 mL. First, for a given preprocedural risk, there were differences along the spectrum of contrast in the OR of AKI by 200 mL increase of contrast volume. For example, for the risk of a creatinine level increase of at least 0.3 mg/dL, the OR for a patient with 5% preprocedural risk with a contrast volume increase of 100 to 300 mL was 1.36 (95% CI, 1.32-1.43) and became 1.64 (95% CI, 1.52-1.77) for a contrast volume increase of 400 to 600 mL (Figure 3A; eTable 4 in the Supplement). Second, there were differences in ORs between preprocedural risk groups by the same contrast increase. For example, for an increase of contrast volume of 100 to 300 mL, patients with a 45% preprocedural risk had the highest OR and RD compared with patients with a 5% or 80% preprocedural risk (eg, risk of ≥0.5 mg/dL increase in creatinine levels: 45% procedural risk, OR, 1.56; 95% CI, 1.48-1.65; RD, 9.7%; 95% CI, 8.5%-10.8%; 5% preprocedural risk, OR, 1.41; 95% CI, 1.33-1.49; RD, 0.4%; 95% CI, 0.3%-0.4%; 80% preprocedural risk, OR, 1.26; 95% CI, 1.06-1.50; RD, 5.3%; 95% CI, 1.3%-9.2%) (Figure 3B; eTable 4 in the Supplement). Third, there were differences between severity levels of AKI in the association of contrast volume with AKI. For example, for patients with a 5% preprocedural risk, the ORs and RDs for an increase of at least 0.5 mg/dL or 1.0 mg/dL had a larger difference from a 100 to 300 mL increase in contrast volume and from a 400 to 600 mL increase in contrast volume than the OR and RD for the risk of a creatinine level increase of at least 0.3 mg/dL (risk of ≥0.3 mg/dL increase in creatinine levels with 100-300 mL increase in contrast volume, OR, 1.36; 95% CI, 1.32-1.42; RD, 1.1%; 95% CI, 1.0%-1.2%; with 400-600 mL increase in contrast volume, OR, 1.64; 95% CI, 1.52-1.77; RD, 3.1%; 95% CI, 2.5%-3.7%; risk of ≥0.5 mg/dL increase in creatinine levels with 100-300 mL increase in contrast volume, OR, 1.41; 95% CI, 1.33-1.49; RD, 0.4%; 95% CI, 0.3%-0.4%; with 400-600 mL increase in contrast volume, OR, 1.97; 95% CI, 1.75-2.21; RD, 1.7%; 95% CI, 1.3%-2.2%; risk of ≥1.0 mg/dL increase in creatinine levels with 100-300 mL increase in contrast volume, OR, 1.51; 95% CI, 1.36-1.67; RD, 0.1%; 95% CI, 0.1%-0.2%; with 400-600 mL increase in contrast volume, OR, 2.27; 95% CI, 1.91-2.70; RD, 0.7%; 95% CI, 0.5%-1.0%) (Figure 3; eTable 4 in the Supplement).

## Discussion

We developed and validated a model for quantifying the association of contrast volume with the risk of 3 AKI severities for patients undergoing PCI. We found that the risk increase varied with the volume of contrast and the patient’s baseline risk. Given our results and prior studies showing little variation in the current use of contrast by patient risk,^2^ the model has the potential to support personalized estimates of contrast dosing to reduce AKI risk.

Previous work on guiding contrast dosing focused on deriving a safe maximum level of contrast volume.^4,20,21^ However, different safe levels were derived from cohorts with different inclusion criteria, posing a challenge for their use in a general population of patients undergoing PCI.^6,21,22^ Moreover, information on AKI risks associated with incremental contrast volume below and above the safe level is valuable for decision-making.^6^ Furthermore, these studies have assumed a linear association between AKI risk and contrast volume, ignoring possible heterogeneity in this association among different patients. In contrast, our model was built on more than 1 million patients across a broad spectrum of baseline risk, making it applicable for a general population of patients undergoing PCI. More importantly, the advanced analytics used in developing this model allow insight into a more complex association between contrast volume and AKI risk, beyond that acknowledged by the literature. The heterogeneity found in the association between AKI and contrast volume among patients could enable personalized decisions on contrast dosing and could even help to inform the decision to have PCI.

Physicians base the diagnosis of AKI on an elevation in serum creatinine concentration, but there are different definitions of the degree of elevation required for a diagnosis of AKI. Common definitions use an absolute increase above 0.3 mg/dL, 0.5 mg/dL, or 1.0 mg/dL, a relative increase above 25%, or a composite of the absolute and relative increase.^23,24,25,26,27^ These definitions would identify different patients with an AKI diagnosis; thus, prediction models built on 1 definition cannot be applied to other definitions.^28^ To develop a prediction model for guiding contrast dosing that is applicable irrespective of the AKI definition under consideration, we chose to use 3 thresholds of absolute increase in creatinine level as the study outcome, including the 0.3 mg/dL threshold as part of the AKIN definition, the 0.5 mg/dL threshold commonly used in previous studies, and a highest threshold of 1.0 mg/dL. Providing predictions for different severities of AKI would enable decision-making according to the severity level for each patient as determined by the clinician. We did not include the relative changes in our outcome definition because they depend on baseline creatinine level and may introduce discontinuity in the association between outcome and the 2 predictors, resulting in difficulty in modeling. Even though we did not use relative creatinine, it can be calculated for decision-making by using baseline creatinine levels. The derived model simultaneously predicted risks for the spectrum of AKI severity, providing a more nuanced understanding of the association of contrast volume with AKI to guide informed decisions on contrast dosing.

The approach implemented in this study could be applied to other clinical outcomes and modifiable procedural risk factors to investigate the association of procedural variables with the estimate of outcome risk, providing guidance for decisions surrounding risk-mitigation strategies. A large body of work on clinical prediction modeling is based on models that exclusively use baseline information. Such a model is useful for both benchmarking quality assessment and planning treatment.^29^ With the additional integration of procedural variables, there is opportunity to identify new and helpful information for personalized strategies from the data.

Our model quantifying the association between AKI risk and contrast volume for individual patients is a step toward personalized decision-making about the net benefit of PCI and personalized treatment planning for PCI to reduce AKI risk. There are several next steps for implementing this model. First, the current model used registry data, in which some of the data elements required manual abstraction. To integrate the model as decision support at the point of care, there is a need to automate variable extraction, most likely from electronic health records that include clinical notes. This need is increasingly being addressed by advancements in the computational capacities of electronic health records and advanced analytics as active research topics. Second, the effective implementation of the model through integration with clinical workflow to encourage model utility in decision-making also needs to be established. Last, the predictions provided by the model should be considered alongside information on the complexity of the case and the risk profile of the patient. These factors should be presented in an interactive interface for clinicians to make informed decisions.

### Limitations

This study has several limitations. First, the model was developed using observational data on PCI patients who had exposure to contrast. However, the similar patterns in contrast use observed for different baseline risks ensured that the bias owing to contrast use preference conditioned on baseline risk was limited. Second, although the model was temporally validated, it may need to be continually updated to reflect changes in patient characteristics and associations in data.^30^ Third, the model cannot provide predictions for patients with very high preprocedural risks and very high contrast volumes owing to lack of data in the cohort for these combinations. However, such high volume is rarely considered for these patients. In the event that high volume is needed, predictions from the model should not be used for decision support because so few patients with high risk in our database had that exposure. Fourth, we have no information on when the postprocedural creatinine levels were assessed, and patients discharged the same day of the procedure were excluded. These omissions may have failed to recognize AKIs in which creatinine levels rose after discharge. However, these patients are often at the lowest risk for AKI and do not have postprocedural creatinine levels assessed. The model remains applicable for general PCI patients, especially for those with an elevated risk of AKI who could benefit most from the model. Fifth, we do not have access to outcomes after discharge for these patients to evaluate how the identified creatinine level increases were associated with those outcomes. Further study should investigate whether the model could reduce the risk of clinical adverse outcomes. Sixth, this study did not assess types of contrast agents in association with AKI risk, which may be a topic for further investigation.

## Conclusions

We developed a model to estimate the association of contrast volume with AKI risk for patients undergoing PCI. We found this association to be nonlinear and heterogeneous among patients with different baseline risks. The model provided information that could guide personalized contrast dosing for individual patients to reduce their risks of AKI. Further investigation is needed to apply and validate the model at the point of care and evaluate its impact on decision-making and outcomes.

## Notes

Supplement. **eFigure 1. **Study Flow for Model Development and Internal and Temporal Validations **eFigure 2. **Model Selection via Visual Comparison Between Scatterplot of Observations and Contour Plots of Predictions by Candidate Models **eFigure 3. **Calibration Plots of the Model Predicting Risks of Acute Kidney Injury in Deciles in the Test Set **eFigure 4. **Calibration Plots of the Model in Deciles in the Validation Cohort **eFigure 5. **Calibration Plots of the Model via Splines in the Validation Cohort **eFigure 6. **Modeled Risk of Acute Kidney Injury as a Function of Baseline Risk and Contrast Volume **eTable 1. **Predictors in the Machine Learning Model for Preprocedural Risk Estimation **eTable 2. **Use Pattern of Contrast Volume **eTable 3. **Model Performance ComparisonBetween Multinomial and Ordinal Logit Link Functions **eTable 4. **Odds Ratio and Absolute Risk Difference of Acute Kidney Injury by 200 mL Increase in Contrast Volume
