# Supplementary material for: Development and Validation of a Model for Predicting the Risk of Acute Kidney Injury Associated With Contrast Volume Levels During Percutaneous Coronary Intervention
Source: JAMA Netw Open. 2019 Nov 22;2(11):e1916021. doi: 10.1001/jamanetworkopen.2019.16021 (PMC6902830; doi:10.1001/jamanetworkopen.2019.16021)
Supplement: Supplement. — eFigure 1. Study Flow for Model Development and Internal and Temporal Validations eFigure 2. Model Selection via Visual Comparison Between Scatterplot of Observations and Contour Plots of Predictions by Candidate Models eFigure 3. Calibration Plots of the Model Predicting Risks of Acute Kidney Injury in Deciles in the Test Set eFigure 4. Calibration Plots of the Model in Deciles in the Validation Cohort eFigure 5. Calibration Plots of the Model via Splines in the Validation Cohort eFigure 6. Modeled Risk of Acute Kidney Injury as a Function of Baseline Risk and Contrast Volume eTable 1. Predictors in the Machine Learning Model for Preprocedural Risk Estimation eTable 2. Use Pattern of Contrast Volume eTable 3. Model Performance ComparisonBetween Multinomial and Ordinal Logit Link Functions eTable 4. Odds Ratio and Absolute Risk Difference of Acute Kidney Injury by 200 mL Increase in Contrast Volume [file jamanetwopen-2-e1916021-s001.pdf]

Supplementary Online Content

Huang C, Li S-X, Mahajan S, et al. Development and validation of a model for predicting the risk of acute kidney injury associated with contrast volume levels during percutaneous coronary intervention. *JAMA Netw Open*. 2019;2(11):e1916021. doi:10.1001/jamanetworkopen.2019.16021

eFigure 1. Study Flow for Model Development and Internal and Temporal Validations

eFigure 2. Model Selection via Visual Comparison Between Scatterplot of Observations and Contour Plots of Predictions by Candidate Models

eFigure 3. Calibration Plots of the Model Predicting Risks of Acute Kidney Injury in Deciles in the Test Set

eFigure 4. Calibration Plots of the Model in Deciles in the Validation Cohort

eFigure 5. Calibration Plots of the Model via Splines in the Validation Cohort

eFigure 6. Modeled Risk of Acute Kidney Injury as a Function of Baseline Risk and Contrast Volume

eTable 1. Predictors in the Machine Learning Model for Preprocedural Risk Estimation

eTable 2. Use Pattern of Contrast Volume

eTable 3. Model Performance Comparison Between Multinomial and Ordinal Logit Link Functions

eTable 4. Odds Ratio and Absolute Risk Difference of Acute Kidney Injury by 200 mL Increase in Contrast Volume

This supplementary material has been provided by the authors to give readers additional information about their work.

© 2019 Huang C et al. *JAMA Network Open.*

eFigure 1. Study Flow for Model Development and Internal and Temporal Validations

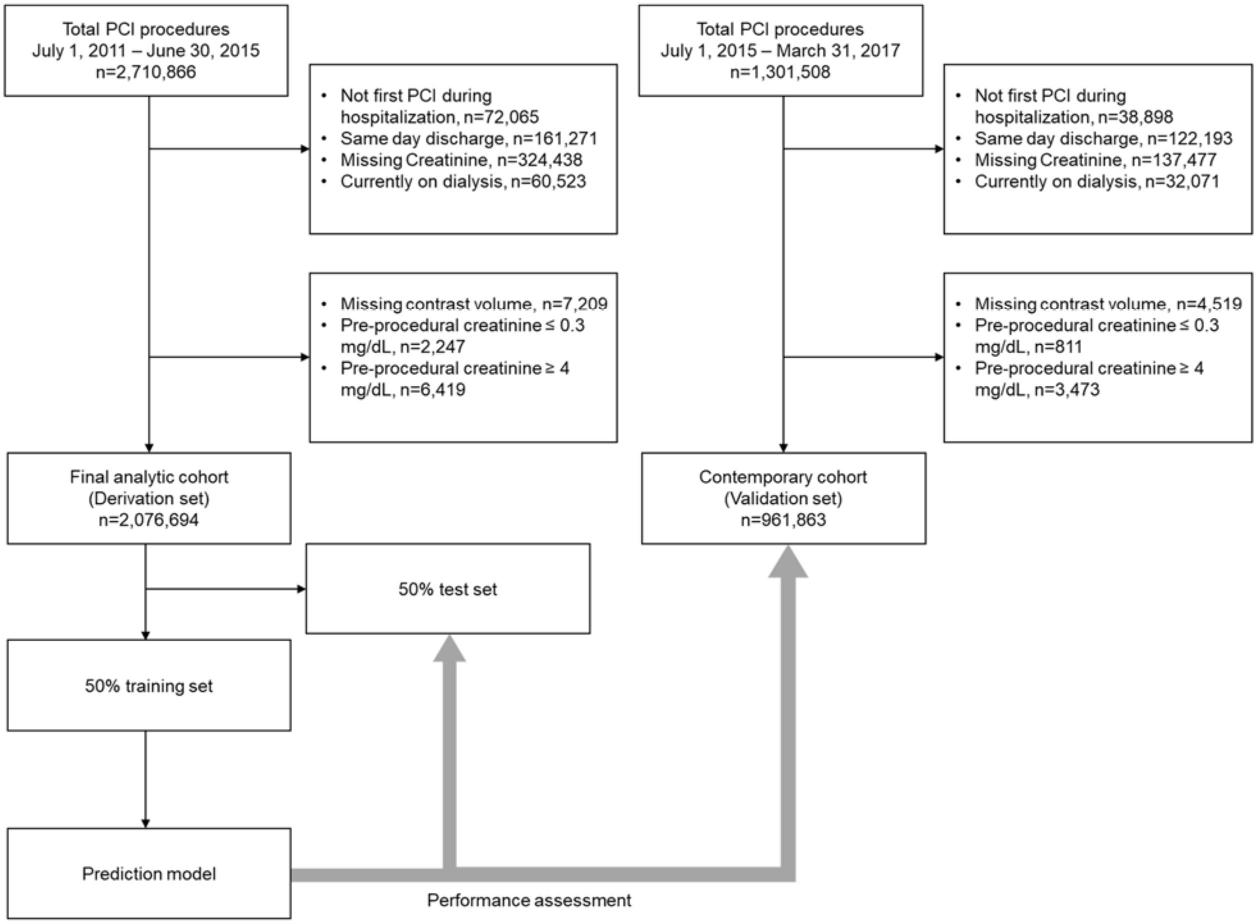

© 2019 Huang C et al. *JAMA Network Open.*

eFigure 2. Model Selection via Visual Comparison Between Scatterplot of Observations and Contour Plots of Predictions by Candidate Models

Comparison of (A) observed risk of creatinine increase at least 0.3 mg/dL to predictions from (B) model with linear variables, (C) model with linear variables and interaction term, (D) model with nonlinear variables, and (D) model with nonlinear variables and interaction term.

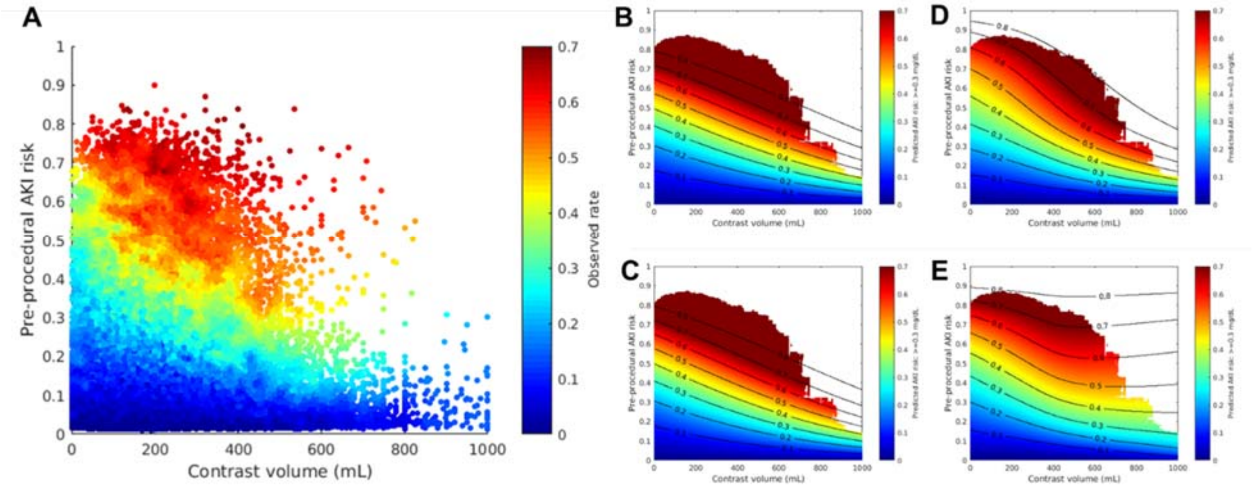

© 2019 Huang C et al. *JAMA Network Open.*

eFigure 3. Calibration Plots of the Model Predicting Risks of Acute Kidney Injury in Deciles in the Test Set

The calibration plots are produced for observed versus deciles of predicted risks of absolute increase in creatinine of (A) 0.3 mg/dL, (B) 0.5 mg/dL, and (C) 1.0 mg/dL were calculated in the observed versus predicted risks via cubic spline smoothing.

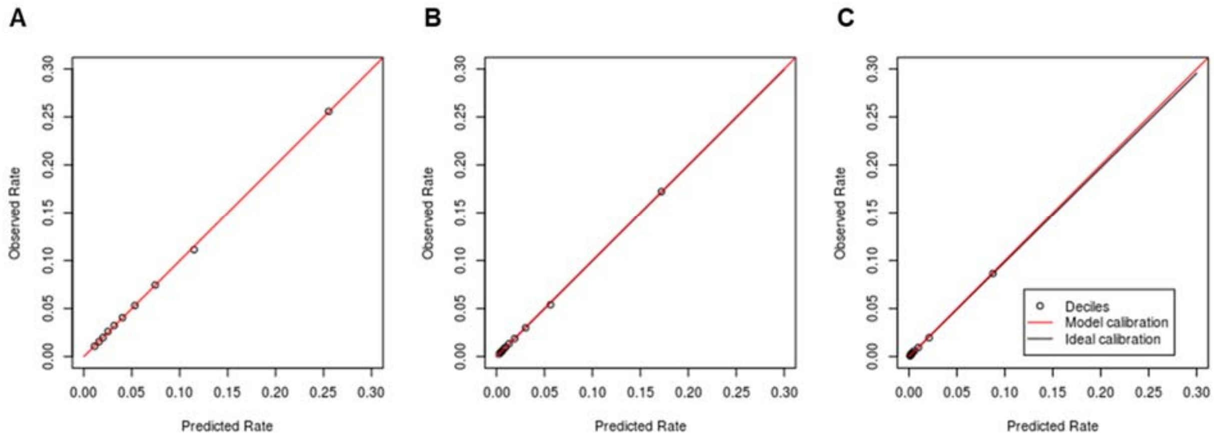

© 2019 Huang C et al. *JAMA Network Open.*

eFigure 4. Calibration Plots of the Model in Deciles in the Validation Cohort

Risks of absolute increase in creatinine of at least (A) 0.3 mg/dL, (B) 0.5 mg/dL, and (C) 1.0 mg/dL were calculated in the observed versus predicted deciles of risks. The black line and the dotted lines indicate the calibration of the model with 95% confidence interval. The red line indicates ideal calibration.

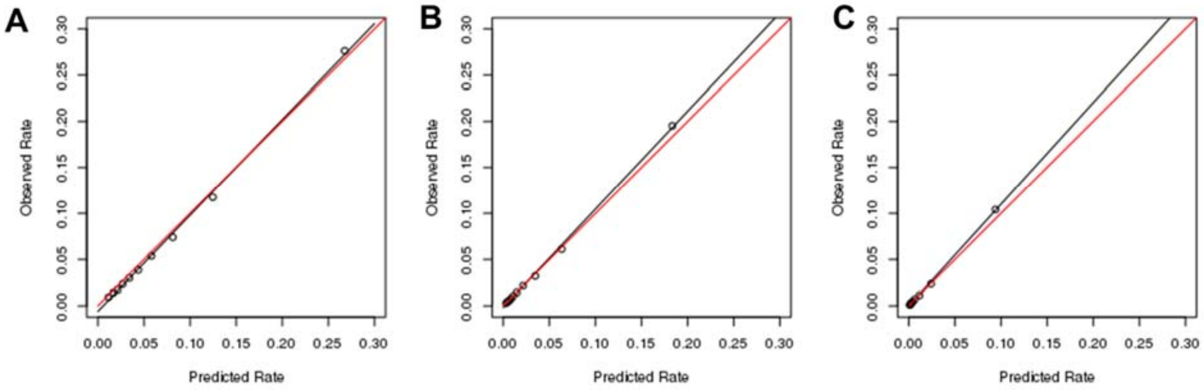

© 2019 Huang C et al. *JAMA Network Open.*

eFigure 5. Calibration Plots of the Model via Splines in the Validation Cohort

Risks of absolute increase in creatinine of at least (A) 0.3 mg/dL, (B) 0.5 mg/dL, and (C) 1.0 mg/dL were calculated in the observed versus predicted risks via cubic spline smoothing. The black line and the dotted lines indicate the calibration of the model with 95% confidence interval. The red line indicates ideal calibration.

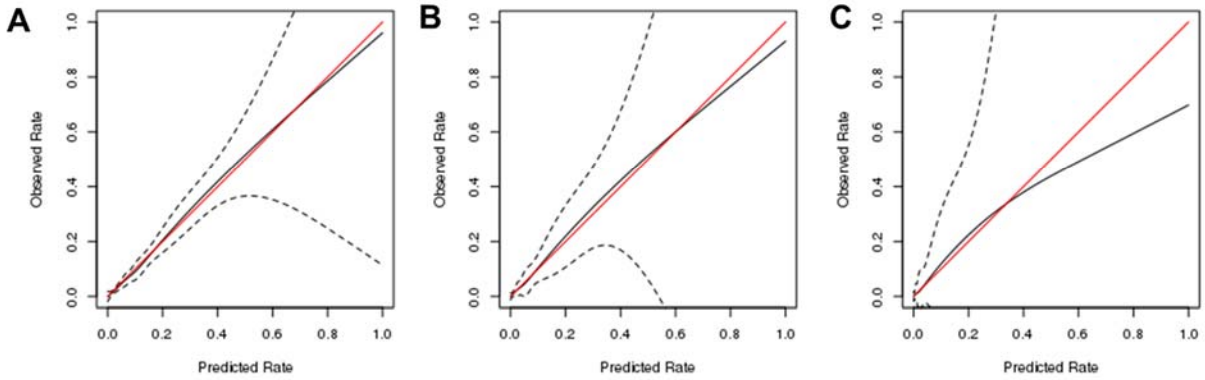

© 2019 Huang C et al. *JAMA Network Open.*

eFigure 6. Modeled Risk of Acute Kidney Injury as a Function of Baseline Risk and Contrast Volume

Risks of absolute increase in creatinine of at least (A) 0.3 mg/dL, (B) 0.5 mg/dL, and (C) 1.0 mg/dL.

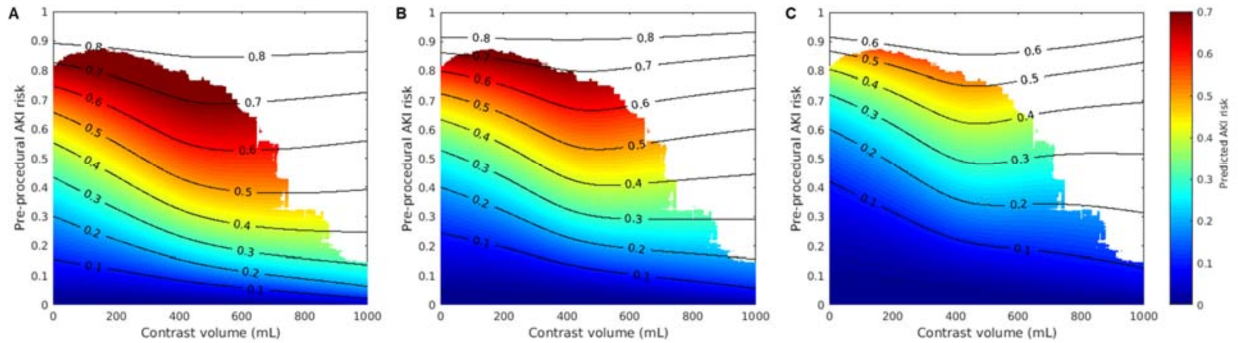

© 2019 Huang C et al. *JAMA Network Open.*

eTable 1. Predictors in the Machine Learning Model for Preprocedural Risk Estimation

**Predictors**	**Coding details**
Age	
Prior heart failure	
Cardiogenic shock w/in 24 hours	no vs. yes
Cardiac arrest w/in 24 hours	no vs. yes
Diabetes mellitus composite	no vs. yes, insulin vs. yes, other therapies
CAD presentation composite	non-STEMI/others
Heart failure w/in 2 weeks composite	no vs. yes, NYHA class IV vs. yes, other classes
Pre-procedure GFR	
Pre-procedure hemoglobin	
Admission source	emergency department vs. other admission sources
Body mass index	
PCI status	elective vs. emergency vs. other statuses
Pre-PCI ventricular ejection fraction	

CAD, coronary artery disease; STEMI, ST-elevation myocardial infarction; NYHA, New York Heart Association; GFR, glomerular filtration rate; PCI, percutaneous coronary intervention

© 2019 Huang C et al. *JAMA Network Open.*

eTable 2. Use Pattern of Contrast Volume

Derivation cohort											
**n (%[a])**	**Contrast volume, mL**										
**Pre-procedural AKI risk, %**	**0–50**	**50–100**	**100–150**	**150–200**	**200–250**	**250–300**	**300–350**	**350–400**	**400–600**	**>600**	**All**
0–5	14,432 (1.4)	101,441 (9.8)	253,306 (24.6)	279,209 (27.1)	181,538 (17.6)	102,956 (10.0)	48,254 (4.7)	25,541 (2.5)	22,153 (2.1)	1,952 (0.2)	1,030,782 (100.0)
5–10	9,954 (1.7)	62,934 (10.7)	145,266 (24.8)	155,853 (26.7)	101,302 (17.3)	56,824 (9.7)	26,510 (4.5)	13,653 (2.3)	11,432 (2.0)	898 (0.2)	584,626 (100.0)
10–25	8,635 (2.4)	46,457 (12.9)	92,486 (25.7)	92,395 (25.7)	58,040 (16.2)	32,236 (9.0)	14,903 (4.1)	7,539 (2.1)	6,363 (1.8)	500 (0.1)	359,554 (100.0)
25–50	3,540 (4.0)	14,821 (16.9)	23,309 (26.5)	20,639 (23.5)	12,396 (14.1)	6,762 (7.7)	3,142 (3.6)	1,600 (1.8)	1,517 (1.7)	119 (0.1)	87,845 (100.0)
50–75	652 (4.8)	2,320 (17.0)	3,392 (24.9)	3,023 (22.2)	1,914 (14.1)	1,132 (8.3)	557 (4.1)	295 (2.2)	296 (2.2)	30 (0.2)	13,611 (100.0)
>75	14 (5.1)	41 (14.9)	64 (23.2)	71 (25.7)	43 (15.6)	21 (7.6)	7 (2.5)	6 (2.2)	6 (2.2)	3 (1.1)	276 (100.0)
**Validation cohort**											
**n (%[a])**	**Contrast volume, mL**										
**Pre-procedural AKI risk, %**	**0–50**	**50–100**	**100–150**	**150–200**	**200–250**	**250–300**	**300–350**	**350–400**	**400–600**	**>600**	**All**
0–5	5,093 (1.2)	49,617 (11.3)	119,642 (27.2)	118,624 (27.0)	72,607 (16.5)	39,395 (9.0)	17,639 (4.0)	9,098 (2.1)	7,340 (1.7)	640 (0.1)	439,695 (100.0)
5–10	4,280 (1.5)	34,648 (12.3)	77,490 (27.5)	74,919 (26.6)	45,471 (16.2)	24,240 (8.6)	10,794 (3.8)	5,417 (1.9)	4,60 (1.5)	318 (0.1)	281,937 (100.0)
10–25	4,409 (2.4)	27,786 (15.0)	52,272 (28.2)	47,269 (25.5)	27,485 (14.8)	14,297 (7.7)	6,342 (3.4)	3,056 (1.6)	2,524 (1.4)	201 (0.1)	185,641 (100.0)
25–50	2,111 (4.5)	8,986 (19.1)	13,292 (28.3)	10,775 (22.9)	6,040 (12.8)	3,130 (6.7)	1,386 (2.9)	723 (1.5)	565 (1.2)	32 (0.1)	47,040 (100.0)
50–75	474 (6.4)	1,438 (19.5)	1,906 (25.9)	1,535 (20.8)	957 (13.0)	537 (7.2)	251 (3.4)	140 (1.9)	111 (1.5)	29 (0.3)	7,530 (100.0)
>75	11 (6.8)	24 (14.8)	39 (24.1)	41 (25.3)	18 (11.1)	8 (4.9)	10 (6.2)	5 (3.1)	6 (3.7)	0 (0.0)	162 (100.0)

[a]Percentage is calculated row-wise.

© 2019 Huang C et al. *JAMA Network Open.*

eTable 3. Model Performance Comparison[a] Between Multinomial and Ordinal Logit Link Functions

	≥0.3 mg/dL	≥0.5 mg/dL	≥1.0 mg/dL
Event rate	6.4%	3.2%	1.4%
**Multinomial logit link**			
AUC	0.778 (0.776,0.780)	0.839 (0.837,0.841)	0.870 (0.867,0.873)
Calibration slope	1.002 (0.993,1.011)	1.005 (0.995,1.015)	1.006 (0.994,1.020)
Calibration intercept	-0.000 (-0.001,0.000)	-0.000 (-0.001,0.000)	-0.000 (-0.000,0.000)
Brier score	0.0541 (0.0540,0.0542)	0.0278 (0.0278,0.0279)	0.0125 (0.0125,0.0126)
Predictive range	24.6% (24.4%,24.8%)	17.1% (17.0%,17.3%)	8.8% (8.7%,8.9%)
**Ordinal logit link**			
AUC	0.778 (0.776,0.780)	0.839 (0.837,0.841)	0.870 (0.867,0.873)
Calibration slope	0.949 (0.940,0.958)	1.186 (1.175,1.197)	1.346 (1.329,1.363)
Calibration intercept	0.002 (0.002,0.003)	-0.007 (-0.007,-0.007)	-0.005 (-0.005,-0.005)
Brier score	0.0541 (0.0540,0.0543)	0.0279 (0.0279,0.0280)	0.0126 (0.126,0.126)
Predictive range	24.6% (24.4%,24.9%)	17.1% (16.9%,17.3%)	8.8% (8.7%,8.9%)

[a]Performance was evaluated on training set for prediction risks of absolute increase in creatinine of at least 0.3 mg/dL, 0.5 mg/dL,and 1.0 mg/dL.

AUC, area under the receiver operating characteristic curve

© 2019 Huang C et al. *JAMA Network Open.*

eTable 4. Odds Ratio and Absolute Risk Difference of Acute Kidney Injury[a] by 200 mL Increase in Contrast Volume

**≥0.3 mg/dL**	**100–300 mL**		**400–600 mL**		**700–900 mL**	
**Pre-procedural AKI risk**	**OR**	**RD, %**	**OR**	**RD, %**	**OR**	**RD, %**
5%	1.36 (1.32–1.42)	1.1 (1.0–1.2)	1.64 (1.52–1.77)	3.1 (2.5–3.7)	1.51 (1.30–1.76)	4.5 (2.4–6.6)
45%	1.56 (1.49–1.64)	10.7 (9.6–11.9)	1.15 (0.98–1.34)	3.4 (0–7.3)	-[b]	-
80%	1.30 (1.10–1.54)	5.2 (1.9–8.6)	-	-	-	-
**≥0.5 mg/dL**	**100–300 mL**		**400–600 mL**		**700–900 mL**	
**Pre-procedural AKI risk**	**OR**	**RD, %**	**OR**	**RD, %**	**OR**	**RD, %**
5%	1.41 (1.33–1.49)	0.4 (0.3–0.4)	1.97 (1.75–2.21)	1.7 (1.3–2.2)	1.63 (1.30–2.04)	2.8 (1.0–4.6)
45%	1.56 (1.48–1.65)	9.7 (8.5–10.8)	1.1 (0.89–1.28)	1.6 (0–6.1)	-	-
80%	1.26 (1.06-1.50)	5.3 (1.3-9.2)	-	-	-	-
**≥1.0 mg/dL**	**100–300 mL**		**400–600 mL**		**700–900 mL**	
**Pre-procedural AKI risk**	**OR**	**RD, %**	**OR**	**RD, %**	**OR**	**RD, %**
5%	1.51 (1.36–1.67)	0.1 (0.1–0.2)	2.27 (1.91–2.70)	0.7 (0.5–1.0)	1.88 (1.36–2.61)	1.6 (0.3–3.0)
45%	1.75 (1.64–1.87)	8.3 (7.3–9.3)	1.03 (0.82–1.30)	0.6 (0–5.1)	-	-
80%	1.38 (1.12–1.69)	8.0 (2.8–13.1)	-	-	-	-

AKI, acute kidney injury; OR, odds ratio; RD, absolute risk difference.

[a]Risk of absolute increase in creatinine of at least 0.3 mg/dL, 0.5 mg/dL, and 1.0 mg/dL.

[b]OR and RD were not calculated if there were <10 patients in the neighborhood of the values of pre-procedural AKI risk and contrast volume.

© 2019 Huang C et al. *JAMA Network Open.*
